# Incretin and insulin signaling as novel therapeutic targets for Alzheimer’s and Parkinson’s disease

**DOI:** 10.1038/s41380-022-01792-4

**Published:** 2022-10-18

**Authors:** Joseph Nowell, Eleanor Blunt, Paul Edison

**Affiliations:** 1 Division of Neurology, Department of Brain Sciences, Imperial College London, London, UK; 2 School of Medicine, Cardiff University, Cardiff, UK

**Keywords:** Drug discovery, Neuroscience

## Abstract

Despite an ever-growing prevalence and increasing economic burden of Alzheimer’s disease (AD) and Parkinson’s disease (PD), recent advances in drug development have only resulted in minimally effective treatment. In AD, along with amyloid and tau phosphorylation, there is an associated increase in inflammation/glial activation, a decrease in synaptic function, an increase in astrocyte activation, and a state of insulin resistance. In PD, along with α-synuclein accumulation, there is associated inflammation, synaptic dysfunction, dopaminergic neuronal loss, and some data to suggest insulin resistance. Therapeutic strategies for neurodegenerative disorders have commonly targeted individual pathological processes. An effective treatment might require either utilization of multiple drugs which target the individual pathological processes which underlie the neurodegenerative disease or the use of a single agent which could influence multiple pathological processes. Insulin and incretins are compounds with multiple effects on neurodegenerative processes. Preclinical studies have demonstrated that GLP-1 receptor agonists reduce neuroinflammation, reduce tau phosphorylation, reduce amyloid deposition, increase synaptic function, and improve memory formation. Incretin mimetics may act through the restoration of insulin signaling pathways, inducing further neuroprotective effects. Currently, phase 2 and phase 3 trials are underway in AD and PD populations. Here, we provide a comprehensive review of the therapeutic potential of incretin mimetics and insulin in AD and PD.

## Introduction

Alzheimer’s disease (AD) and Parkinson’s disease (PD) are the two most common neurodegenerative diseases [1]. AD is characterized by a progressive loss of cognitive function, memory impairment, and decline in activities of daily living [2]. There are currently around 54 million patients with a diagnosis of dementia worldwide with incidence rates predicted to triple internationally to 152 million people by 2050 and will cost health services $2 trillion a year by 2030 as per the World Alzheimer Report 2018 [3]. Pathologically, AD is characterized by extracellular ß-amyloid (Aß) plaques, and intracellular neurofibrillary tangles (NFTs). Apart from Aß plaques and NFTs, other pathological features of AD include neuroinflammation and microglial activation, synaptic dysfunction, reduced cerebral glucose metabolism, astrocyte activation, and neuronal loss [4]. PD is characterized by bradykinesia, rigidity, and tremor [5]. Non-motor symptoms of PD are often equally as debilitating and include cognitive dysfunction and neurological defects [6]. In 2016, the prevalence of PD was 6.1 million globally, with a conservative estimate for numbers to double by 2050 [7].

While treatment strategies for AD have principally targeted individual pathological hallmarks such as amyloid and tau with limited success in modifying the disease course, to have an effective disease-modifying treatment it may be necessary to target multiple pathologies [8, 9]. Drugs that target inflammation, synaptic dysfunction, insulin resistance, and oxidative stress, all of which lead to neuronal death and neurodegeneration, should also be considered for an effective therapeutic strategy for AD and PD. In this review, we focus on how GLP-1 receptor agonists, insulin and other incretin mimetics exert multiple neuroprotective effects and could provide an effective new therapeutic strategy for neurodegenerative diseases.

## GLP-1

By activating an incretin signaling pathway, glucagon-like peptide-1 (GLP-1) and glucose-dependent insulinotropic polypeptide (GIP) modulate blood glucose levels [10]. Within the central nervous system (CNS) GLP-1 is produced within the nucleus of the solitary tract, intermediate reticular nucleus, piriform cortex, and the olfactory bulb, which activate GLP-1 receptors [11–13]. GLP-1 receptors are expressed in several nuclei within the brainstem, hypothalamus, and some limbic areas [14]. It is also possible that GLP-1 receptors within the brain are activated by circulating GLP-1 produced in the periphery [14]. Ligand binding initiates several downstream signaling cascades which initiate cell growth and repair. Native GLP-1 has a short half-life of 1–2 min owing to rapid degradation by dipeptidyl peptidase-4 (DPP-4) [15]. GLP-1 receptor agonists resistant to DPP-4 degradation such as semaglutide, albiglutide, and dulaglutide have been developed, generating an extended half-life in circulation [15]. GLP-1 receptor agonists have been used successfully and safely in type 2 diabetes mellitus (T2DM) and do not induce insulin desensitization after prolonged use, as they do not activate insulin receptors in normoglycemic subjects [16] and thus do not cause hypoglycemia.

**Fig. 1:**
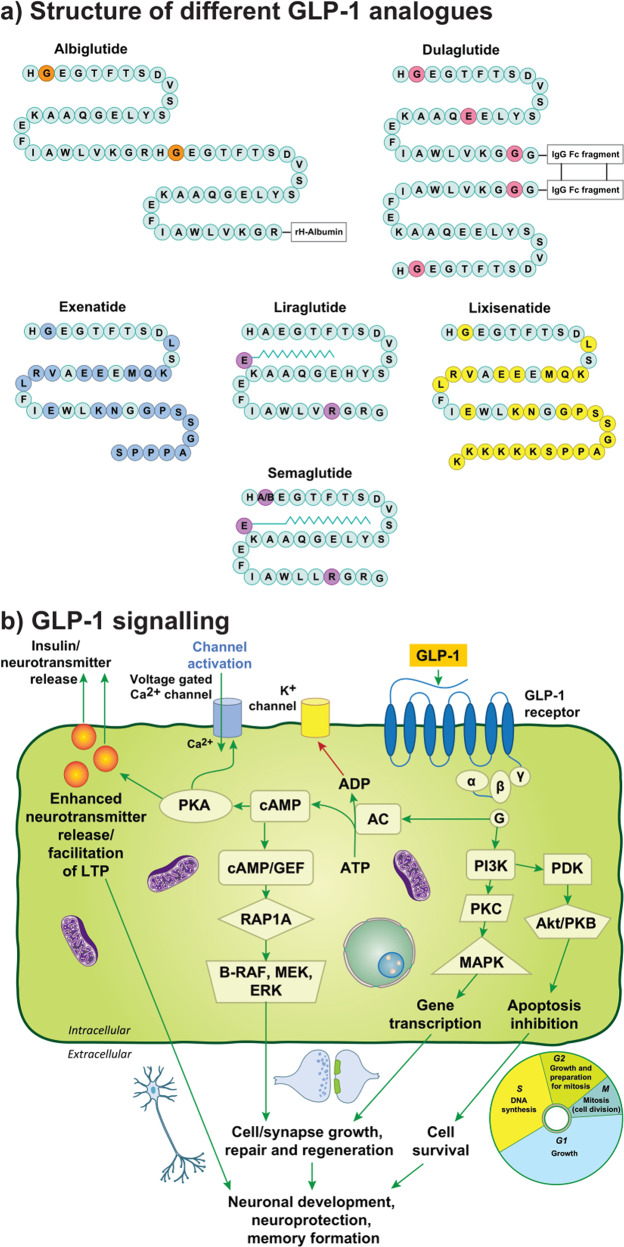
GLP-1 structure and cerebral cell signaling. **a** shows structure of GLP-1 analogues exenatide, lixisenatide, liraglutide, albiglutide, semaglutide and dulaglutide. **b** displays downstream signaling pathways from GLP-1 activation. Abbreviations: Fc fragment crystallizable, GLP-1 glucagon-like peptide-1, IgG immunoglobulin G, AC adenylyl cyclase, ADP adenosine diphosphate, Akt murine thymoma viral oncogene homologue, B-Raf B-regulation of alpha-fetoprotein, cAMP cyclic adenosine monophosphate, ERK extracellular signal-regulated kinase, GEFs guanine-nucleotide-exchange factors, GLP-1 glucagon-like peptide-1, GLP-1R glucagon-like peptide-1 receptor, LTP long-term potentiation, MAPK mitogen-activated protein kinases, MEK MAPK kinase, PDK phosphatidylinosite dependent kinase, PI3K phosphatidylinositol- 4,5-bisphosphate 3-kinase, PKA protein kinase A, PKB protein kinase B, PKC protein kinase C, Rap1A Ras-related protein Rap-1A.

Exendin-4 is a peptide agonist of the GLP-1 receptor which was discovered in the saliva of the Gila monster, *Heloderma suspectum* [17]. The synthetic version of exendin-4, exenatide, shares a 53% amino acid sequence homology with human GLP-1 [18] and has been investigated in treating PD. Exenatide was the first in the class of GLP-1 receptor agonists approved and is widely prescribed for T2DM [19]. Exendin-4 has been shown to cross the blood-brain barrier (BBB) at high doses in mice [20], and exenatide demonstrates the fastest rates of brain influx compared to GLP-1 receptor agonists lixisenatide, liraglutide, and semaglutide [21]. Lixisenatide is also a short-acting GLP-1 receptor agonist based on the structure of exendin-4 with a half-life of approximately 3 hours [22]. Liraglutide is another GLP-1 receptor agonist, most commonly used either as mono- or combined therapy. Liraglutide has 97% homology with human GLP-1 with a half-life of over 13 hours [23]. Regarding the properties of liraglutide to cross the BBB, in patients with T2DM minimal transfer from blood to CSF was identified [24]. Semaglutide, albiglutide and dulaglutide are other DPP-4 resistant GLP-1 receptor agonists [25]. Albiglutide has a half-life of 120 h and dulaglutide has a half-life of 90 h, therefore administration is only required once a week [26]. Semaglutide has an extended half-life of 160 h and offers a significant advantage since it is also available as a daily oral formulation. Gabery et al. reported that semaglutide accessed the brainstem, hypothalamus, and the dorsal half of the septal area, largely via small circumventricular organs, but it was not shown to cross the BBB [27]. The central distribution of semaglutide and liraglutide was similar, however, only semaglutide was observed within the lateral septal nucleus [27, 28]. Evidence for whether GLP-1 receptor agonists can penetrate the BBB remains limited as it has been argued that brain capillary binding or sequestration may not have been accounted for [29]. A recent investigation compared brain uptake of several labeled incretin receptor agonists in rodents [21]. Single receptor agonists, liraglutide and semaglutide, were not shown to measurably cross the BBB, implying that these GLP-1 receptor agonists may influence brain function indirectly and act at brain regions outside the BBB [21]. Exenatide and lixisenatide were demonstrated to cross the BBB, suggesting a significant advantage of these GLP-1 receptor agonists in search for a treatment for AD and PD [21]. (See Fig. 1a. for an overview of GLP-1 receptor agonist structures).

### GLP-1 receptor agonists in AD

GLP-1 receptor agonists exert their effect on neuronal function by multiple mechanisms. These include reducing inflammation, diminishing tau phosphorylation, and improving synaptic function, in addition to their influence on insulin resistance [30].

#### Possible mechanism

GLP-1 receptor binding can trigger several pathways through activation of specific G subunits which include Gα and Gβγ subtypes [31]. The GLP-1 receptor coupled Gα subunit (Gαs) pathway activates the adenylate cyclase (AC) system [32]. AC system activation generates an increase of the intracellular secondary messenger, cyclic adenosine monophosphate (cAMP), through the conversion of adenosine triphosphate, which then results in the activation of protein kinase A (PKA) and exchange protein activated by cAMP (Epac) [32–35]. Subsequently, PKA activity can augment the release of neurotransmitters into the synapse, facilitating long-term potentiation (LTP) and promoting cAMP-response element binding protein (CREB) phosphorylation, supporting cell growth/survival as well as synaptic plasticity [31, 33]. Key substrates are phosphorylated by PKA, which are involved in insulin synthesis and secretion [31]. The metabolic ligand adenosine diphosphate (ADP), which derives from the AC, acts on K^+^ channels, increasing membrane potential following channel closure and reducing the repolarization stage [33]. The resultant opening of voltage-dependent L-type Ca^2+^ channels increases the intracellular concentration of Ca^2+^, which also acts as a second messenger, augmenting neurotransmitter release [33]. Separate signaling cascades can also determine an increase of cytosolic Ca^2+^ which includes the phosphorylation of PKA after GLP-1 receptor activation, the increase of phosphatidylinositol-4,5-bisphosphate 3-kinase (PI3K) levels and the mobilization of Ca^2+^ stores [33, 36]. PI3K is activated by the GLP-1 receptor coupled Gβγ subunits which consequently activate mitogen-activated protein kinases (MAPK), initiating gene expression that controls cell growth, repair, and differentiation [33, 37]. A further signaling cascade implicated includes cAMP binding and activation of Rap1A and guanine nucleotide exchange factors (GEFs) [31–34]. (Fig. 1b).

GLP-1 receptor agonists exert their effect by binding to GLP-1 receptors in multiple tissues and organs. GLP-1 receptors are present in the brain, cerebral blood vessels, pancreas, heart, gastrointestinal tract, adipose tissue, kidney, and muscles, and consequently affect a variety of systems and processes [38–40]. In the heart, GLP-1 decreases systolic blood pressure and promotes cardioprotection. The link between GLP-1 and the heart also highlights the link between dementia and cardiometabolic disease, whereby risk reduction can pose as an additional effective mechanism in the treatment of AD [41]. GLP-1 has a vasodilatory action and anti-atherosclerotic effect [42]. A protective effect of GLP-1 agonists on blood vessels has been demonstrated, as evidenced by the reduction of non-fatal and fatal stroke incidence by 15% and 19%, respectively, in patients with T2DM treated with GLP-1 agonists [43]. Activation of GLP-1 receptors in cortical arterioles enhances cerebral blood flow and improves perfusion, which may promote neuroprotection against ischemic stroke [44].

#### Preclinical evidence

In vivo studies utilizing the APP/PS1 mouse model of AD indicated neuroprotective properties of liraglutide. Synaptic loss, cognitive symptoms such as memory impairment, and inflammation shown by activated microglia, were either alleviated or reversed [45, 46]. This was further supported by experiments in the SAMP8 mouse model of AD, in which liraglutide delayed or halted the decline in memory function along with hippocampal neuronal loss [47]. It has also been shown that liraglutide reduced Aβ synthesis, including reduction of dense core plaque formation [45, 46]. Duarte et al. revealed that liraglutide partially normalized PKA-mediated signaling in female 3xTg-AD mice. The authors also identified that liraglutide treatment reduced central inflammation and oxidative stress [48]. In addition, liraglutide reversed cognitive impairment in mice and attenuated insulin receptor and synaptic pathology in a non-human primate model of AD [49]. Recently Xie et al. evaluated the influence of GLP-1 in 5xFAD mice, where liraglutide restored astrocytic mitochondrial function and prevented neuronal loss. In vitro, GLP-1 enhanced brain-derived neurotrophic factor (BDNF) secretion and ameliorated mitochondrial dysfunction and cell toxicity via cAMP/PKA activation in Aβ-treated astrocytes [50]. Liraglutide treatment in 5xFAD mice improved spatial memory, an effect associated with enhanced aerobic glycolysis in astrocytes which improved cell support, promoting neuronal survival [51]. Furthermore, in 3-month-old transgenic 5xFAD mice, liraglutide reduced astrocyte activation and Aβ deposition while increasing levels of insulin-degrading enzyme (IDE) [52].

Several additional GLP-1 receptor agonists also display neuroprotective properties in preclinical AD models. It has been shown that exendin-4 reduces mitochondrial toxicity in an Aβ-induced AD mouse model, an effect that was mediated through PI3K/protein kinase B (Akt) signaling [53]. This was supported in 5xFAD mice, in which exendin-4 treatment alleviated cognitive dysfunction, prevented amyloid-β accumulation, and protected mitochondrial function [54]. In SH-SY5Y cell cultures, semaglutide was shown to protect against Aβ_25-35,_ through enhancement of autophagy and inhibition of apoptosis [55]. A recently developed GLP-1 receptor agonist with a long half-life, CJC-1131, was effective in reversing cognitive impairments, restoring hippocampal LTP, and protecting against Aβ toxicity [56]. Furthermore, GLP-1 receptor agonists protect against neurodegeneration by suppressing tau hyperphosphorylation. Hansen et al. demonstrated that liraglutide reduced pathology-specific tau phosphorylation and improved motor function in a transgenic hTauP301L mouse model of tauopathy [57].

#### Clinical evidence

In a small-scale, double-blind study, 38 AD patients were randomly assigned to receive either liraglutide or placebo [58]. Whilst treatment failed to reduce amyloid plaque load, liraglutide did prevent the decline in cerebral glucose metabolism after 26 weeks compared to placebo treatment, which suggests that liraglutide may modify disease progression. Another placebo-controlled trial assessed the influence of 12-week liraglutide treatment on functional connectivity in mid-aged individuals with subjective cognitive impairment [59]. Inclusion criteria included a mini-mental state exam score >27, suggesting intact cognition, with 32 subjects completing study procedures, half of whom had a family history of AD. Liraglutide treatment prevented functional decline within the default mode network structures in contrast to placebo, further suggesting that GLP-1 receptor agonists can exert neuroprotective effects in AD and provide a promising disease-modifying strategy. However, no cognitive improvement was identified, which probably reflects the limited statistical power and short treatment period of the trial to identify meaningful differences. The trial Evaluating Liraglutide in Alzheimer’s Disease (ELAD) recently completed a 12-month phase 2b clinical trial of liraglutide in 204 AD patients, awaiting publication [60]. Initial clinical findings suggest that GLP-1 receptor agonists could provide an effective therapeutic strategy in patients with AD and those at risk, however, there remains a lack of sufficient evidence in large clinical samples to draw any firm conclusions. Semaglutide is entering phase 3 evaluation in patients with early AD (NCT04777396).

The REWIND trial, targeting cardiovascular events in T2DM participants without previous cardiovascular history, found that dulaglutide reduced cardiovascular outcomes, and was well tolerated [61]. In an exploratory analysis of REWIND, the hazard of substantive cognitive impairment was reduced by 14% in the dulaglutide group [62]. One possible confounding factor in translating the results to AD involves the detrimental influence of hyperglycemia on cognition [63, 64]. The REWIND trial included patients with hemoglobin A1c levels of up to 9.5%, thus the influence of dulaglutide on glycemic control may underlie the reduced risk of cognitive dysfunction. A *post-hoc* analysis of pooled data from three cardiovascular outcome trials in 15,820 T2DM patients promisingly revealed treatment with liraglutide or semaglutide resulted in a two-fold reduction in the risk of being diagnosed with dementia compared to placebo [65]. Despite appearing promising, as the prevalence of diabetes in patients with dementia is around 13–20% [66] whether the findings from diabetic patients apply to the wider AD population is debatable.

### GLP-1 receptor agonists in PD

Approved treatments for PD target dopaminergic neurotransmission, with levodopa representing the mainstay of treatments augmenting dopamine production and alleviating motor symptoms [6]. However, serious side effects frequently arise following prolonged administration of levodopa, including dyskinesia, and there are currently no treatments available that affect disease progression.

#### Possible mechanism

**Fig. 2:**
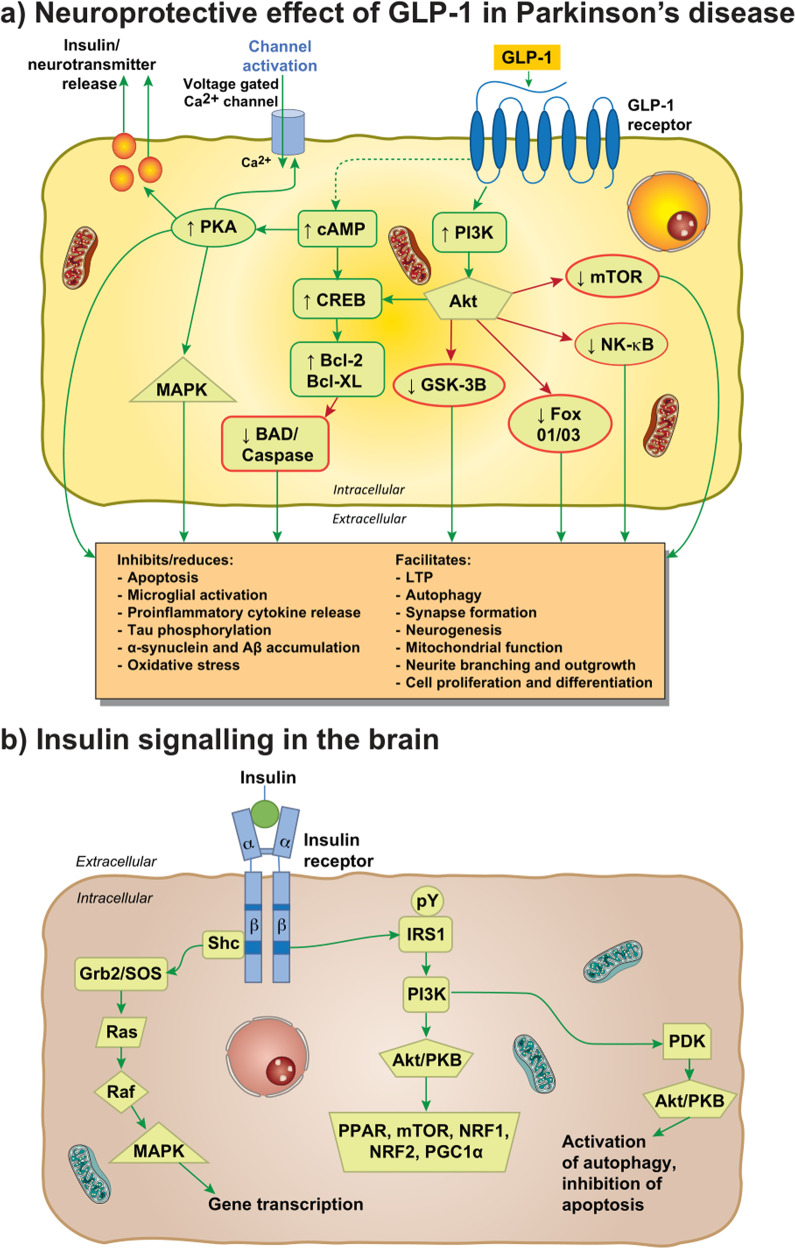
Neuroprotective effects of GLP-1 and insulin signaling in the brain. **a** shows downstream signaling pathways depicting the influence GLP-1 on Parkinson’s disease pathogenesis. **b** Insulin signaling in neurons. **b** demonstrates the action of insulin in the neuron and activation of downstream pathway. Abbreviations: Bcl-2 B cell lymphoma 2, BAD (Bcl-2) antagonist of death, Bcl-XL B cell lymphoma 2 extra-large, cAMP cyclic AMP, CREB cAMP response element-binding protein, FoxO1/O3 Forkhead box O1/O3, GLP-1 glucagon-like peptide-1, GSK-3B glycogen synthase 3 beta, LTP long-term potentiation, MAPK mitogen-associated protein kinase, mTOR mammalian target of rapamycin, NF-kB nuclear factor kappa-light-chain-enhancer of activated B cells, PI3K phophoinositide 3-kinase, TNF tumor necrosis factor, Akt/PKB protein kinase B complex, CPD3B cyclic phosphodiesterase 3 beta, Grb2/SOS Growth factor receptor binding protein 2/son of sevenless protein, IRS insulin receptor substrates that get phosphorylated after activation, NRF1 nuclear respiratory factor-1, PGC-1α peroxisome proliferator-activated receptor γ coactivator 1-α, PDK phosphatidylinosite dependent kinase, PPAR peroxisome proliferator-activated receptor family, Raf regulation of alpha-fetoprotein, Ras rat sarcoma virus peptide, Shc Src homology collagen peptide.

Inflammation [67], oxidative stress [68], and apoptosis [69] are implicated in PD pathogenesis. There are some data to suggest that insulin resistance also occurs in PD [70], however, this is controversial since systemic insulin resistance is not present in de novo, medication-free PD patients [71], and brain insulin resistance is not present in cognitively normal PD cases [72]. GLP-1 influences PD pathogenesis via its G protein-coupled receptor, which activates both cAMP-PKA pathways and PI3Ks pathways acting via Akt, PKC, and MAPK (Fig. 1b). The involvement of GLP-1 in PD can be simplified to the activation of the MAPK/extracellular signal-regulated kinase 1/2 (ERK) pathway, which has an important role in synaptic plasticity, and the PI3K/Akt pathway signaled by GLP-1 receptor stimulation and the subsequent increase in cAMP and PKA and PI3K activation [73]. The AKT pathway can phosphorylate more than fifty substrate proteins and modulate processes disrupted in PD, such as the enhancement of synapse formation, autophagy, and LTP, as well as inhibiting the secretion of pro-inflammatory cytokines, apoptosis, microglial activation, tau phosphorylation and the accumulation of α-synuclein and Aβ (Fig. 2a). Thus, GLP-1 signaling represents a promising target for PD treatment. GLP-1 receptor activation can reduce neuroinflammation and oxidative stress, and restore insulin signaling, which may compensate for the dysfunctional dopaminergic neurotransmission and motor deficits associated with PD pathogenesis [74, 75]. Recent research indicates additional neuroprotective processes via GLP-1-mediated enhancement of BBB integrity [76], which requires further evaluation.

#### Preclinical evidence

Perry et al. observed that, in vitro, exendin-4 may promote neurite outgrowth via both the ERK MAPK and the PI3K signaling pathways and may be able to rescue degenerating neurons [77]. Furthermore, exendin-4 administration in a 1-methyl-4-phenyl-1,2,3,6-tetrahydropyridine (MPTP) in vivo animal model of PD has been shown to inhibit microglial activation thereby minimizing the loss of dopaminergic neurons in the pars compacta of the substantia nigra and the striatum [78]. In support, Li et al. observed the neuroprotective effect of exenatide in both cultured embryonic primary cerebral cortical and ventral mesencephalic (dopaminergic) neurons, while also improving motor activity in MPTP mice [79]. Moreover, peripheral administration of exenatide improved motor function in an in vivo adult 6-hydroxydopamine model of PD, possibly via neurogenesis [80]. Liraglutide and semaglutide have shown efficacy in reducing the α-synuclein load in the chronic MPTP mouse model of PD [81]. Beneficial effects of GLP-1 in reducing toxic protein accumulation are hypothesized to occur through the PI3K/Akt inactivation of glycogen synthase kinase 3 beta (GSK3β) [82] and a possible GLP-1 mediated upregulation of IDE [83]. In vivo and in vitro models of PD demonstrated that exendin-4 ameliorated behavioral deficits and reduced α-synuclein pathology, and enhanced autophagy via restoration of PI3K/Akt/mammalian target of rapamycin (mTOR) signaling [84]. GLP-1 receptor agonists, liraglutide and lixisenatide, also display neuroprotective properties in MPTP mouse models; both GLP-1 receptor agonists were superior in reducing motor impairments compared to exenatide [85]. Taken together, preclinical models of PD demonstrate the promising neuroprotective potential for GLP-1 receptor agonists to reduce inflammation and α-synuclein accumulation, whilst restoring mitochondrial function and motor deficits.

#### Clinical evidence

The 2013 exenatide proof-of-concept clinical trial involving 45 moderate PD patients displayed possible efficacy concerning part 3 of the Movement Disorders Society Unified Parkinson’s Disease Rating Scale (MDS-UPDRS) [17]. Exenatide-treated patients had a mean 2.7 point improvement on the MDS-UPDRS, compared to a mean decline of 2.2 points in controls. The advantages observed from this proof-of-concept trial continued 12 months after exenatide cessation [17, 86]. In a follow-up proof-of-concept study by Aviles-Olmos et al., the longevity of motor and non-motor effects were assessed up to 24 months after the trial. Cognitive scores, measured by the Mattis DRS-2, depicted a 5-point advantage at 12 months, a 6.3 advantage at 14 months, and a 5.3-point advantage at 24 months of exenatide compared to controls [86]. Thus, exenatide treated patients maintained their cognitive benefit 12-months following drug cessation, however, it should be noted that cognitive scores also remained unchanged for controls between 12 and 24 months. Furthermore, owing to the single-blind design, these results should not be treated as evidence of symptomatic or disease-modifying efficacy of exenatide [17]. In 2017, a single-center, randomized, double-blind clinical trial was conducted to evaluate the efficacy of 2 mg once-weekly of exenatide compared with placebo in patients with PD. The authors found significant improvements in part 3 of the MDS-UPDRS at 48 weeks and 60 weeks after treatment [87]. An exploratory *post-hoc* analysis of trial results suggested that those with milder disease exhibited a better response to exenatide. Additionally, patients who were insulin-resistant or obese at baseline had improved cognitive outcomes following exenatide treatment [88].

In vivo exploration of neuronal-derived extracellular vesicles represents a promising technique to determine the mechanism of action in centrally-acting drugs [89]. Athauda et al. evaluated serum extracellular vesicles as part of a secondary analysis [90], expanding on the positive primary clinical trial outcomes [87]. Exenatide treatment was associated with a prolonged increase of phosphorylation of insulin receptor signaling substrate-1 (IRS1) phosphorylated at tyrosine residues at 24, 48, and 60 weeks. The treatment group also exhibited significant increases in the expression of downstream substrate mTOR after 48 weeks and Akt after 48 and 60 weeks, but not MAPK. The motor improvements observed in exenatide-treated patients were associated with levels of mTOR, indicating that PI3K-Akt signaling mediates the neuroprotective influence of exenatide. Restoration of Akt and mTOR signaling may exert neuroprotective effects, which include increased cell survival [91], a reduction of inflammation [92], and protection of dopaminergic neurons [93]. However, results from Athauda et al. were limited by the fact that their data were restricted to basal level elevations of insulin signaling molecules, as opposed to acute insulin stimulation. Chronic elevation in insulin signaling molecules is a feature of insulin resistance [94], thus it is possible that results reflect insulin signaling dysfunction in participants treated with exenatide.

Exenatide was also safe and well-tolerated, with no patients in the exenatide-treated group withdrawn due to excessive weight loss and weight loss reversed following drug cessation, within the latest trial [87]. However, it remains important to consider that GLP-1 receptor agonists may be limited by unfavorable side effects, especially in the context of PD. GLP-1 can cause a reduction in weight and appetite, with gastrointestinal side effects associated with greater weight loss [95]. Weight loss is a frequent symptom of PD progression, which can be severe with rapid changes in body weight [96].

Phase 2 clinical trials are currently ongoing assessing additional GLP-1 agonists, lixisenatide (NCT03439943), liraglutide (NCT02953665), and semaglutide (NCT03659682) for the treatment of PD [97]. Alongside liraglutide, semaglutide has been shown to effectively improve motor impairments, rescue tyrosine hydroxylase (TH) levels, reduce α-synuclein accumulation, chronic inflammation response in the brain and lipid peroxidation, inhibit mitochondrial mitophagy signaling and protect dopaminergic neurons in the substantia nigra and striatum in MPTP mouse models of PD [81].

### GIP-GLP receptor co-agonists

Alongside GLP-1, GIP is a primary incretin hormone that has shown neuroprotective properties in mouse models of PD and AD [98]. The GIP receptor is a seven-transmembrane G protein-coupled receptor strongly expressed on islet β cells, which has been found in many tissues including the pancreas, adipose tissue, heart, gastrointestinal tract, adrenal cortex, and various neurons in the brain [99]. Both GLP-1 and GIP have been shown to regulate appetite and enhance memory formation with a high expression of GIP in neurons within the hippocampus, olfactory bulb, and Purkinje cells of the cerebellum [100]. Molecular mechanisms downstream of the GIP and GLP-1 receptors have considerable overlap, both exerting insulinotropic effects [101]. GIP receptor signaling increases cAMP levels in the brain, which activates PKA and EPAC [102]. Additionally, GIP receptor signaling activates PI3K, MAPK, and phospholipase A2 [103]. The anti-apoptotic functions of GIP are largely mediated by the PKA-dependent phosphorylation of CREB, whilst the influence of GLP-1 on apoptosis requires PI3K [101, 104]. Knockout of the GIP receptor blocks LTP potentiation in the CA1 area of the hippocampus and induces synaptic dysfunction in mice [105].

GIP receptor agonists have shown neuroprotective properties in mouse models of PD and AD. In an APP/PS1 model of AD, D-Ala2-GIP reduced activation of chronic inflammatory response in the brain, oxidative stress, synapse loss, amyloid plaque burden, and DNA damage [106]. Similar effects have been observed in MPTP mouse models of PD with D-Ala2-GIP-glu-PAL reducing inflammation, oxidative stress, lipid peroxidation, and α-synuclein levels, whilst protecting dopaminergic neurons in the substantia nigra [107]. Treatment also improved motor activity and increased BDNF, which promotes neuroplasticity and protection [108].

Subsequently, dual receptor agonists of GIP and GLP-1 have displayed promising effects in animal models of AD and PD. Dual receptor agonists can activate both GIP and GLP-1 receptors. Utilizing fluorescein-labeled peptide, analysis of dual receptor agonists, exendin-4 and liraglutide, identified that agents with higher BBB permeability have improved protective effects, with DA5‐CH showing the highest efficacy [109, 110]. However, owing to the lack of consideration for brain capillary binding or sequestration, the reliability of these results regarding brain uptake pharmacokinetics has been questioned [21] as well as whether results could be translated into humans. In the APP/PS1 mouse model of AD, dual agonists DA-CH3, DA-CH5, and DA4-JC rescued or prevented spatial learning and memory dysfunction [111–113]. DA-CH5 reduced amyloid plaques and phosphorylated tau [112], while DA-CH3 reduced amyloid plaque as well as resolving endoplasmic reticulum stress and derailed autophagy [111]. DA-CH5 has also been found to reverse deficits in hippocampal late-phase long-term potentiation, upregulate p-PI3K and p-Akt growth factor kinases, and prevent overactivation of p-GSK3β [112].

In PD MPTP animal models, similar neuroprotective effects have been observed. The greatest neuroprotective effects were seen by the use of DA4-JC and DA-CH5 which improve motor function, protect dopaminergic neurons, reduce inflammation, and rescue glial cell line-derived neurotrophic factor levels [114]. When compared to GLP-1 receptor agonists alone, DA-CH5 had greater neuroprotection than exendin-4 [110], whilst DA-CH3 protected dopaminergic neurons, reduced inflammation, and MPTP-induced motor impairment to a greater extent than liraglutide [115]. In a rotenone-induced PD model in rodents, DA4-JC also alleviated motor symptom dysfunctions, protecting dopaminergic neurons mediated through the restoration of mitochondrial function via the Akt/c-Jun N-terminal kinase (JNK) signaling pathway [116]. DA-CH5 effectively reduced α-synuclein, while restoring levels of BDNF and reducing the levels of pro-inflammatory cytokines in MPTP mice [117]. DA4-JC perhaps deserves special consideration in the treatment of AD/PD as it has been shown to cross the BBB effectively, superior to DA3-CH [21].

Importantly, alongside an enhanced neuroprotective effect, dual receptor agonists may have a more balanced influence on weight loss than single receptor agonists [118]. As such, GIP/GLP receptor co-agonists perhaps represent more suitable candidates in the treatment of neurodegenerative conditions.

### GLP-1, GIP, and glucagon receptors agonist

Triple receptor agonists activating GLP-1, GIP, and glucagon receptors have recently been developed. In AD mouse models, pathophysiological changes, such as anti-apoptotic effects, reduced Aβ deposition and phosphorylated hippocampal tau, protection from synapse loss, reduced inflammatory and oxidative stress response in the cortex and hippocampus, and increased BDNF have been observed [119–121]. Furthermore, pre-synaptic and post-synaptic proteins, synaptophysin and PSD-95, which reflect synaptic disruption [122], were upregulated, neuronal excitability normalized, and intracellular calcium, a key regulator in metabolic dysfunction and progressive neuronal loss [123], was modulated [121]. Treatment also rescued long-term memory dysfunction in spatial maze tasks [119–121]. In chronic PD mice, the triple receptor agonist HM15211 protected against dopaminergic neuronal death, decreased α-synuclein accumulation, and prevented nigrostriatal neurodegeneration by mitigating the inflammatory response and lipid peroxidation [124, 125]. MPTP-induced motor impairments were also alleviated.

Despite appearing promising, it remains unclear whether the positive effects of triple agonists differ from those seen in dual GLP-1/GIP agonists. Both triple and dual receptor agonists show similar enhancement of cAMP production in comparison to single GLP-1 receptor agonists [75]. However, recently, a superior effect of triple agonists has been indicated, with triple agonists providing a greater neuroprotective benefit against glutamate excitotoxicity compared with dual receptor agonists [126]. As a result, further research is required into whether triple agonists are beneficial in neurodegenerative populations.

## Role of insulin in AD and PD

Insulin is a hormone primarily secreted by pancreatic β cells and acts as a key factor for cell growth and repair and regulates glucose/lipid metabolism. Insulin exerts its biological effect through interaction with the insulin receptor [127]. This peptide hormone is well known for its mediative effect in regulating cellular glucose transport in the periphery and has been demonstrated to cross the BBB through insulin transporters [128] to act on insulin receptors which are widespread throughout the brain, localized on neurons, astrocytes, and microglia [129–131]. Insulin binds with high affinity to the α-subunits in the extracellular domain of the insulin receptor. Binding to the α-subunits activates the tyrosine kinase activity in the β-subunits, initiating autophosphorylation of the receptor in both neuronal and glial cells. Phosphorylation occurs on various cellular substrates on tyrosine residues including the IRS family and Src homology/collagen (SHC)-transforming family of proteins that mediate signals from the tyrosine kinases [132]. The subsequent downstream signaling cascade initiated from substrates of the insulin receptor activates kinases and transcription factors vital in promoting cell metabolism, neuronal growth and differentiation, gene transcription, synaptic plasticity, and neuroprotection [133] (Fig. 2b). Attenuation of insulin signaling can occur via serine and threonine phosphorylation of IRS1 [134].

Insulin resistance can be defined as a reduced sensitivity to the action of insulin within target tissue [135]. This can be associated with the development of neurodegenerative disorders [136]. Considerable evidence indicates that insulin signaling abnormalities, both peripherally and centrally, are associated with AD and cognitive decline [137]. Alterations in insulin and insulin growth factor-1 (IGF-1) signaling are apparent in AD, causing significant impairments to secondary messenger cascades instigated by the action of insulin [138]. Post-mortem evaluation of hippocampal formation and cerebral cortex tissue demonstrates dysfunctional insulin signaling in AD with elevated basal levels of IRS1 serine phosphorylation and reduced insulin-stimulated levels of downstream signaling molecules [94]. Altered IRS1 phosphorylation has also been demonstrated in both cross-sectional and longitudinal analysis of neuronal-derived exosomes in AD cases in comparison to matched controls [139, 140]. Centrally, IRS1 dysfunction is associated with cognitive decline in AD [141]. In DM, AD, and PD, chronic inflammation may play a significant role in the pathogenesis [142]. Activation of pro-inflammatory tumor necrosis factor-α signaling in response to toxic Aβ oligomers in AD leads to the inhibition of IRS1 [143]. Pro-inflammatory cytokines increase neuronal vulnerability to apoptosis and oxidative stress by activating enzymes such as JNK, ERK, dsRNA-dependent protein kinase (PKR), and IB kinase (IKK) that reduce insulin sensitivity by serine phosphorylation of IRS1 [144, 145]. However, the temporal dynamics and mechanisms underlying insulin resistance development in AD remain unclear. The emergence of abnormal insulin signaling in AD is likely to be multifaceted and involve complex processes [146].

Dysfunctional insulin signaling itself has also been suggested to affect the expression and metabolism of AD-typical pathology—Aβ and tau protein [147]. Neuronal insulin resistance may reduce the levels of IDE, which has an important function in Aβ degradation [148]. IDE cleaves Aβ at multiple sites, reducing the neurotoxicity and accumulation of these proteins [149]. Exposure to insulin is indicated to facilitate the removal of Aβ in astrocytes through increased expression of IDE [150]. Thus, abnormalities in CNS insulin signaling pathways may inhibit Aβ clearance, increasing neurotoxicity and promoting AD. Besides Aβ, insulin signaling disturbance also induces tau protein phosphorylation through the activation of the GSK3β enzyme pathway [151].

While AD is at the forefront of research into the use of insulin in neurodegenerative disease, there is increasing attention being paid to the role of insulin signaling in PD. In vivo studies, such as that by Morris et al., used a preclinical model of a high-fat diet in infant rats to mimic insulin resistance, identifying impaired nigrostriatal dopamine function as a consequence—a hallmark of PD neuropathology [152]. Similarly, insulin resistance displayed by the db/db mouse model of T2DM [153] contributed to α-synuclein accumulation in the midbrain and substantia nigra [154]. These findings translate to clinical research, as increased insulin resistance in patients with PD has been related to the severity of non-motor symptoms [155]. In an attempt to uncover the direct relationship between insulin resistance and the PD etiology, recent research investigated the effects of insulin resistance in a MitoPark mouse model of PD in vitro, in vivo, and ex vivo [156]. Insulin resistance exacerbated pathological PD features which contributed to disease progression via increases in α-synuclein accumulation in dopaminergic neurons, oxidative stress, and mitochondrial dysfunction. A high prevalence of insulin resistance was identified in a sample of 154 non-diabetic PD patients, with 58% being insulin-resistant as indicated by abnormal Homeostatic Model Assessment for Insulin Resistance (HOMA-IR) scores [157]. Dysfunctional brain insulin signaling has been shown using blood-neuron-derived extracellular vesicles, whereby PD patients display altered IRS1 phosphorylation, which is further correlated with tremor severity [158]. Despite the link, the role of insulin abnormalities in PD remains contentious, with little difference found between patients with PD and healthy controls in peripheral insulin resistance, quantified using a hyperinsulinemic-euglycemic clamp [71]. In the only direct evaluation of brain insulin resistance in PD, insulin signaling abnormalities were identified in the dorsolateral prefrontal and posterior parietal cortex of those with cognitive impairment, but not in those who were cognitively normal [72]. Whilst the pathogenesis of PD remains unclear, impaired insulin signaling appears detrimental in the development and progression of PD-related neurodegeneration.

Research evaluating the utility of insulin therapy for AD and PD is underway. Delivery of insulin directly to the CNS is possible through intranasal devices, which, in early trials, has been shown to improve cognition compared to placebo in AD patients [159]. In a pilot trial evaluating 104 amnestic MCI and early-stage AD patients, 20 IU and 40 IU doses of intranasal insulin improved Alzheimer’s Disease Assessment Scale-Cognitive subscale (ADAS-Cog 12) scores compared to placebo-treated patients, with improvements in delayed recall observed 2 months following cessation of treatment in the 20 IU group. Intranasal insulin treatment also prevented the decline in cerebral glucose metabolism [160]. Owing to the promising results, Craft et al. conducted a large-scale, 12-month, phase 2/3 evaluation of 40 IU intranasal insulin per day in 289 patients with a diagnosis of MCI or AD [161]. In this study, insulin treatment failed to improve any primary or secondary outcomes at 12 months and following a 6-month open-label period. Although early trials evaluating the efficacy of insulin showed promise, unfortunately, this result in a larger sample of AD patients indicated that intranasal insulin was insufficient to provide symptomatic or disease-modifying benefits. It is possible that the failure to identify positive results was due to an early change in delivery device to a newer one not yet tested in AD. Patients treated with device 1 showed a near 6-point improvement in performance on ADAS-Cog 12 and CSF Aβ and tau ratios at 18 months, indicating the importance of the intranasal delivery device [159]. A trial evaluating 60 patients with MCI or probable AD discovered that treatment with 40 IU of the long-acting insulin analog, insulin detemir, improved cognition [162]. Results were modulated by the apolipoprotein E (ApoE) genotype. Improved memory was observed in ApoE ε4, whilst worsening memory performance was identified in ApoE ε4 non-carriers. Alongside AD, recently the efficacy of daily administration of 40 IU intranasal insulin was evaluated in PD [163]. In a 4-week pilot trial, verbal fluency scores increased in the group treated with intranasal insulin and scores decreased in the placebo group, but there were no significant paired comparisons identified, reflecting the limited sample size. Motor performance and functionality improvements were seen, with a lower disability score observed in the intranasal insulin group compared to baseline scores, suggesting that intranasal insulin may be a feasible treatment for PD. Several trial limitations, including the small sample size, limit the interpretation of these preliminary findings, which require validation in a larger trial. (Refer to Table 1 for details of clinical trials evaluating GLP-1 and insulin).

**Table 1:** Clinical trials assessing the utility of GLP-1 receptor agonists and insulin in the treatment of AD and PD.

	Target type/name	Trial phase	Population	Key outcomes
*Alzheimer’s*				
Gejl et al., 2016	GLP-1 analogue, liraglutide	26-week, randomized, placebo-controlled, double-blind intervention	38 patients with AD *N* = 18 liraglutide *N* = 20 placebo	Significant decline in placebo group in cerebral glucose metabolism in cingulate and occipital lobes compared to liraglutide treated group after 26 weeks
Watson et al., 2019	GLP-1 analogue, liraglutide	12-week, randomised, placebo controlled trial	26 mid-aged (45–70 years) subjects with subjective cognitive complaints and a Mini-Mental Status Exam score of >27 *N* = 15 liraglutide *N* = 11 placebo	Significant increases in functional connectivity in liraglutide-treated group between the bilateral hippocampus and the left middle frontal gyrus, bilateral posterior cingulate, and the left lateral occipital cortex
Craft et al., 2012	Intranasal insulin	4-month randomized, double-blind, placebo-controlled trial	104 adults with amnestic MCI (*N* = 64) or mild to moderate AD (*N* = 40) *N* = 30 placebo 30 IU *N* = 36 insulin 20 IU *N* = 38 insulin 40 IU	20 IU insulin improved delayed memory. Both doses of insulin preserved caregiver-rated functional ability and reduced the progression of glucose hypometabolism
Craft et al., 2020	Intranasal insulin	12-month phase II/III, randomized, double-blind clinical trial with 6-month open-label extension	240 participants with amnestic MCI or AD (aged 55–85 years) *N* = 121 Intranasal insulin *N* = 119 placebo	No cognitive or functional benefits (early change in the delivery device due to unreliable performance of device 1)
*Parkinson’s*				
Aviles-Olmos et al., 2013	GLP-1 analogue, exenatide	12-month proof-of-concept, single-blind trial design	45 patients with moderate PD *N* = 21 exenatide *N* = 24 controls	Improvements in motor symptoms measured by MDS-UPDRS following exenatide treatment
Athauda et al., 2017	GLP-1 analogue, exenatide	48-week single-center, randomized, double-blind, placebo-controlled trial	62 patients with moderate PD (aged 25–75 years) *N* = 32 exenatide *N* = 30 placebo	Improvements in motor symptoms measured by MDS-UPDRS at 48 weeks and following 12-week washout in the exenatide-treated group
Novak et al., 2019	Intranasal insulin	4-week proof-of concept, randomized, double-blind, placebo-controlled trial	15 patients with a clinical diagnosis of PD or multiple system atrophy *N* = 9 intranasal insulin *N* = 6 placebo	Improved verbal fluency and reduced PD severity in the group treated with intranasal insulin compared with placebo

ApoE ε4 has been demonstrated to impair cerebral insulin signaling by binding to the insulin receptor and preventing insulin receptor trafficking [151]. Thus, acute treatment may be insufficient to provide clinical improvements in ApoE ε4 carriers, with long-lasting stimulation necessary to observe functional benefits [151]. Whereas acute insulin treatment may benefit ApoE ε4 non-carriers with normal insulin levels, chronic treatment may, in fact, induce insulin resistance [151] perhaps explaining a decline in memory function in response to insulin detemir [162]. Gender may also influence beneficial treatment response to intranasal insulin, with dose-dependent differences [164]. Perhaps the optimal dose of insulin selectively benefits different brain regions between men and women [164]. Whilst there is a range of possibilities that would require large-scale investigation, the authors also hypothesize that these discrepancies arise as women are less likely to have insulin abnormalities and are less susceptible to its detrimental effects. As the efficacy of insulin may be modulated by several factors including ApoE genotype and sex, targeted treatment may be necessary.

Despite disappointing results in a large sample of AD patients, insulin therapy merits further evaluation, with a reliable delivery strategy capable of penetrating the BBB, to alleviate cognitive and motor impairments in neurodegenerative disease. Future trials must ascertain the effect of acute and long-acting insulin on cognition whilst accounting for the influence of individual factors, especially ApoE ε4 status. Whilst GLP-1 receptor activation can exert multiple direct neuroprotective effects, the protective influence of GLP-1 receptor agonists may occur indirectly through the restoration of the dysfunctional insulin signaling associated with neurodegenerative disease.

### 4.0 Future therapeutic opportunities in neurodegenerative diseases

Recent clinical and preclinical evidence has established the potential of incretin mimetics to target pathological features central to neurodegenerative disorders. Repurposing GLP-1 receptor agonists and novel dual/triple GLP-1, GIP, and glucagon agonists represent promising therapeutic strategies that have shown to be effective in targeting inflammation, tau phosphorylation, synaptic dysfunction, insulin resistance, and oxidative stress. With many of these pathological processes occurring before the development of clinical symptoms, these agents have the potential to exert their influence in the asymptomatic phase of neurodegenerative disease, but would require further evaluation in preventing neurodegeneration and dementia. The use of GLP-1, GIP, and dual/triple receptor agonists, as well as intranasal insulin, requires further evaluation in large clinical trials. These agents may have the potential to provide disease-modifying treatment for different neurodegenerative disorders. Targeting cerebral incretin and insulin receptors appears to show promise for neurodegenerative conditions, perhaps through neuroprotective mechanisms including reducing cell death and inflammation whilst enhancing neurogenesis.
